# Supplementary figures and images for: Targeting caveolae to pump bispecific antibody to TGF-β into diseased lungs enables ultra-low dose therapeutic efficacy
Source: PLoS One. 2022 Nov 22;17(11):e0276462. doi: 10.1371/journal.pone.0276462 (PMC9681080; doi:10.1371/journal.pone.0276462)

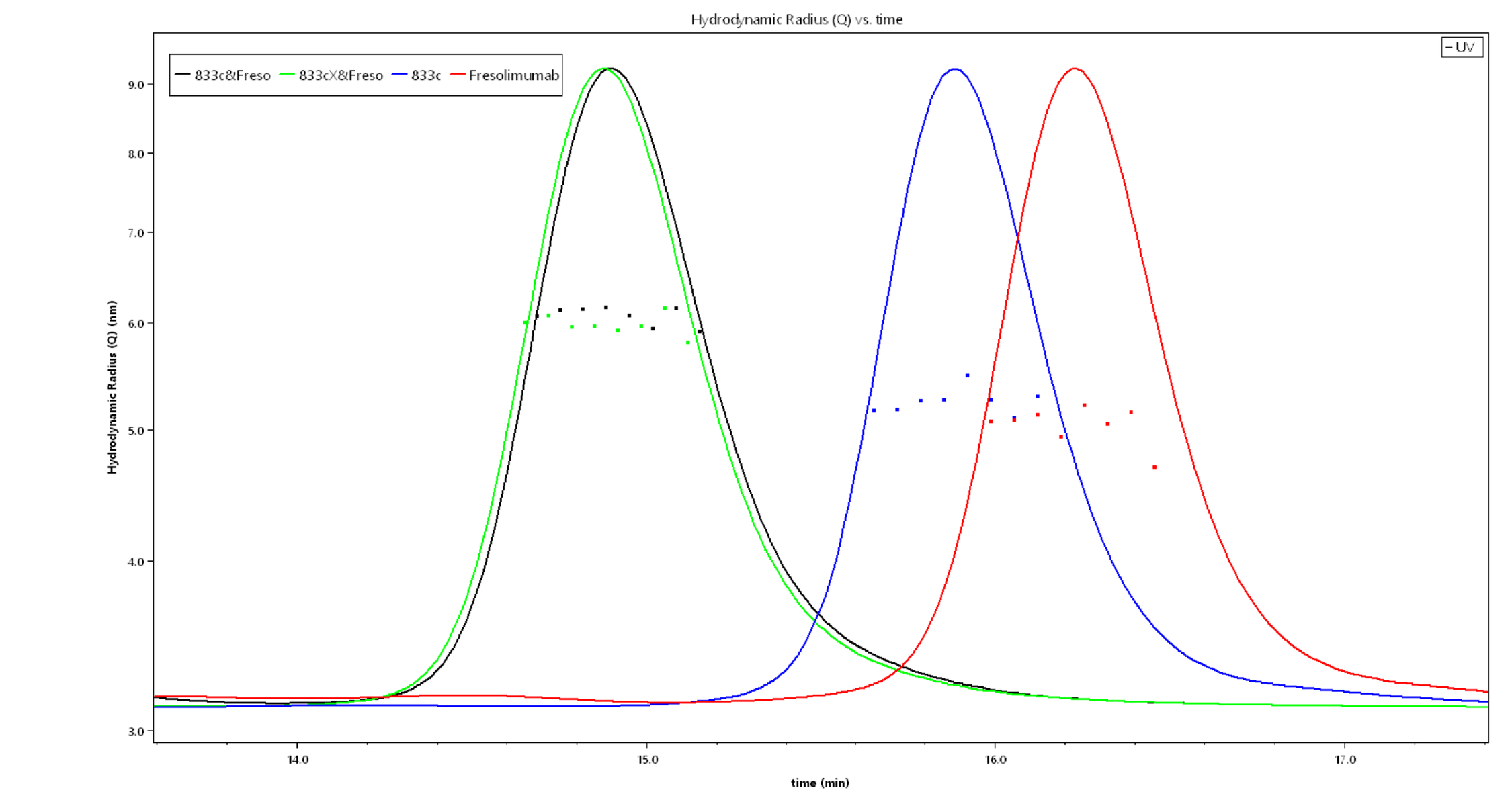

Supplement: S1 Fig — Average hydrodynamic radius (nm): 833c = 5.2, fresolimumab = 5.0, 833c&Freso = 6.1, and 833Xc&Freso = 6.1. (TIF) [file pone.0276462.s001.tif]

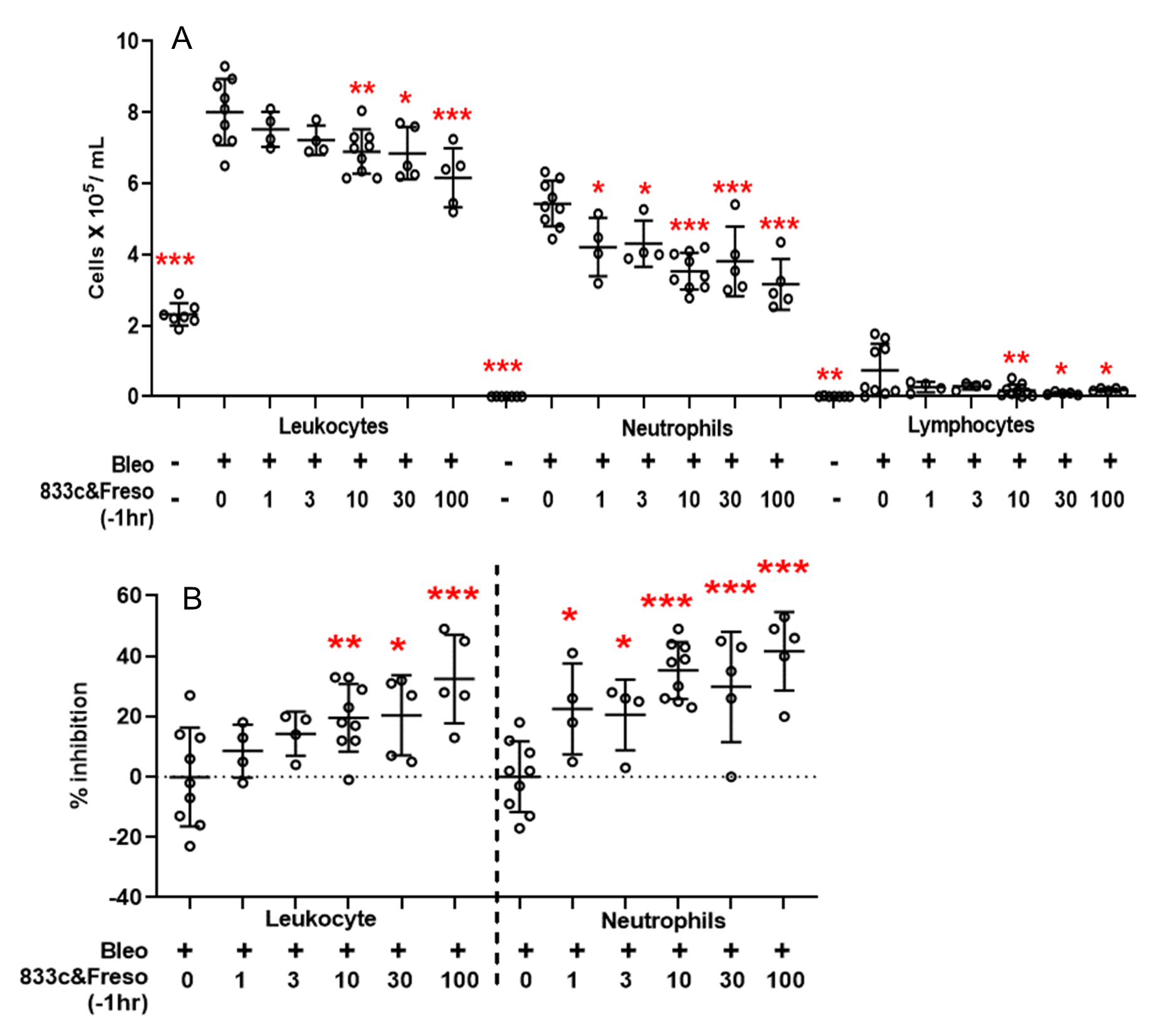

Supplement: S2 Fig — Rats were treated with 833c&Freso at the indicated doses (μg/kg) by iv injection one hour before (-1 h) administering bleomycin (bleo) it (see Methods). BALf was collected on day 3. (A) Total and differential leukocyte concentrations in BALf. (B) Percent (%) inhibition of total leukocyte and neutrophil infiltration. Data were pooled from 2 independent experiments with 833c&Freso 10 μg/kg repeated in both experiments (n = 4–9 total rats per group). Scatter graphs of each data point are shown with indicated means ± SD. *P< 0.03, **P< 0.01, and *** P< 0.001 vs. bleo only, by using one-way ANOVA followed by Dunnett’s test. (TIF) [file pone.0276462.s002.tif]

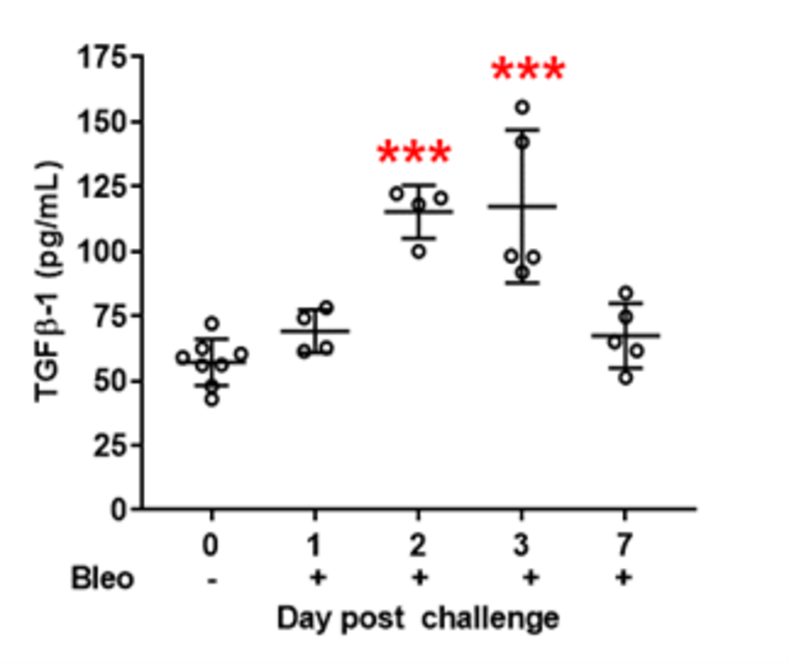

Supplement: S3 Fig — Rats received a single it instillation of bleomycin as described in Methods. BALf was collected and TGF-β1 concentration determined by ELISA. ***P<0.001 vs day 0, by using one way ANOVA followed by Dunnett’s test. (TIF) [file pone.0276462.s003.tif]

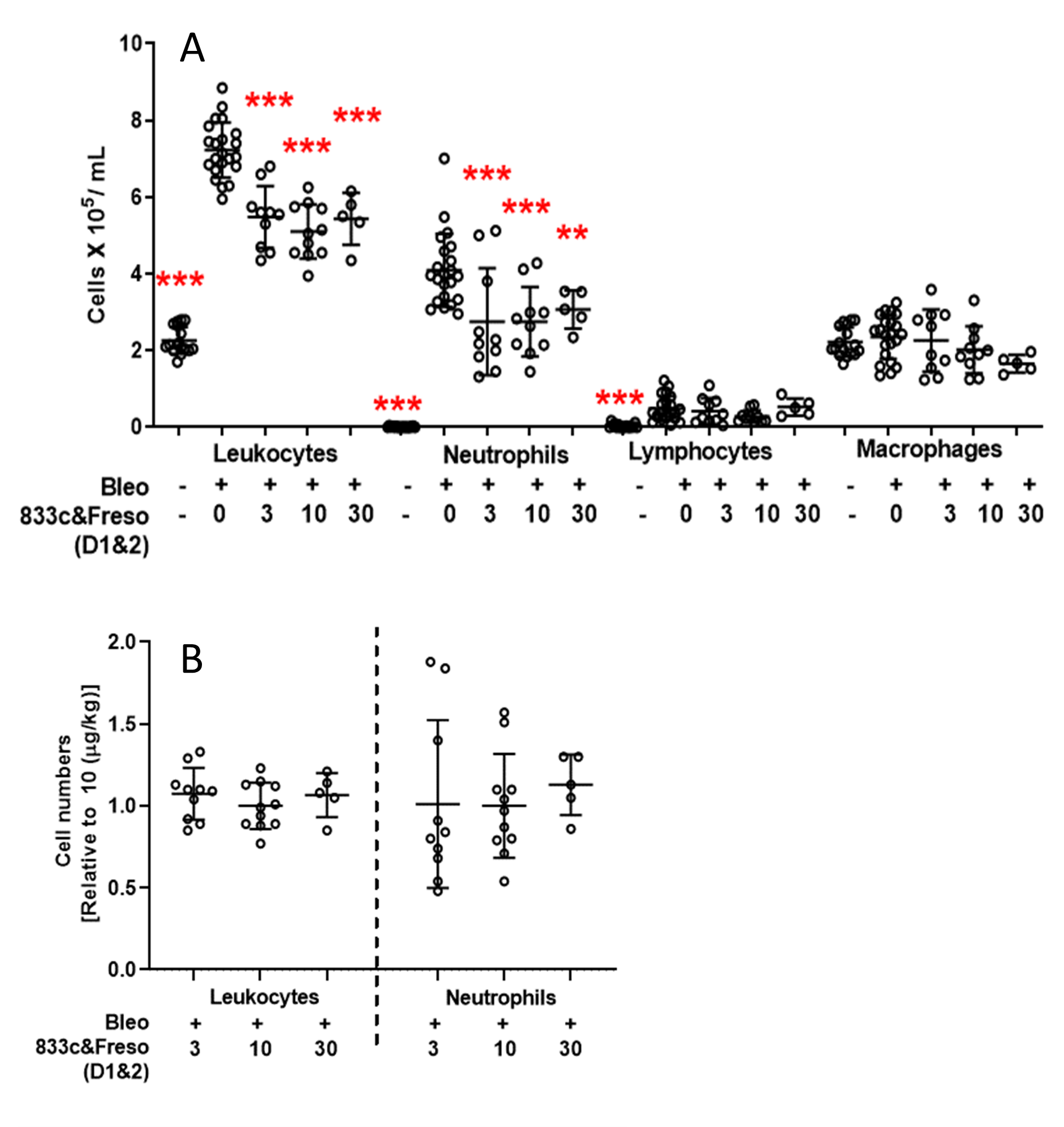

Supplement: S4 Fig — Rats were injected with 833c&Freso at the indicated doses (μg/kg) on day 1 and day 2 (D1&2) after bleomycin (bleo) exposure. BALf was collected on day 3. (A) Total and differential leukocyte profile in BALf. (B) Relative cell numbers normalized to 10 μg/kg. Data pooled from 1–4 independent experiments with n = 5–22 total rats per group. Scatter graphs of each data point with indicated means ± SD. *P< 0.03, **P< 0.01, and *** P< 0.001 vs. bleo only, by using one-way ANOVA followed by Dunnett’s test. (TIF) [file pone.0276462.s004.tif]

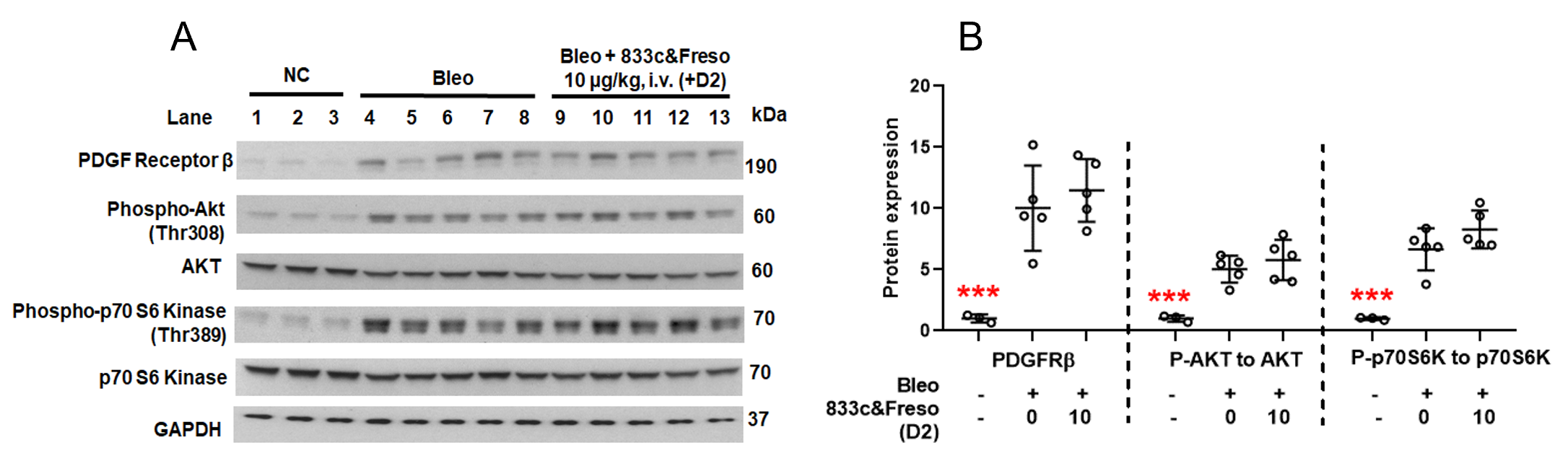

Supplement: S5 Fig — Rats were treated with 10 μg/kg 833c&Freso iv on day 2 post-bleomycin. Lung tissue was harvested on day 3. (A) Lung tissue homogenates were subjected to western blot analysis using antibodies to indicated proteins. (B) Relative expression of PDGFRβ, P-AKT to AKT and P-p70S6K to p70S6K derived from western blot image analysis. Scatter graphs of each data point with indicated means ± SD. N = 3–5 rats per group. *** P< 0.001 vs. bleo only, by using one-way ANOVA followed by Dunnett’s test. (TIF) [file pone.0276462.s005.tif]

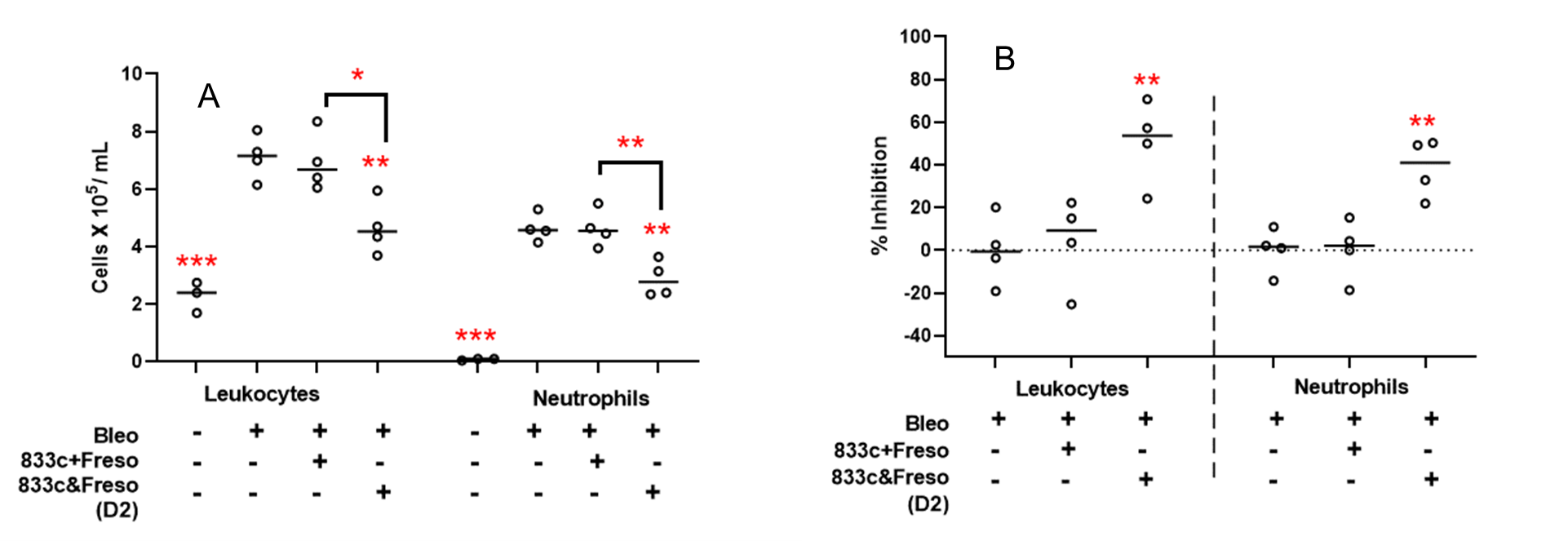

Supplement: S6 Fig — Rats were injected with either10 μg/kg of 833c and fresolimumab or 10μg 833c&Freso on day 2 after bleomycin (bleo) exposure. BALf was collected on day 3. (A) Total leukocyte and neutrophils in BALf. (B) Percentage (%) inhibition of leukocyte and neutrophil infiltration. N = 4 rats per group. Scatter graphs of each data point with indicated means ± SD. *P< 0.03, **P< 0.01, and *** P< 0.001 vs. bleo only, by using one-way ANOVA followed by Dunnett’s test. (TIF) [file pone.0276462.s006.tif]

S1 Fig. Raw image of gel in Fig. 1 D

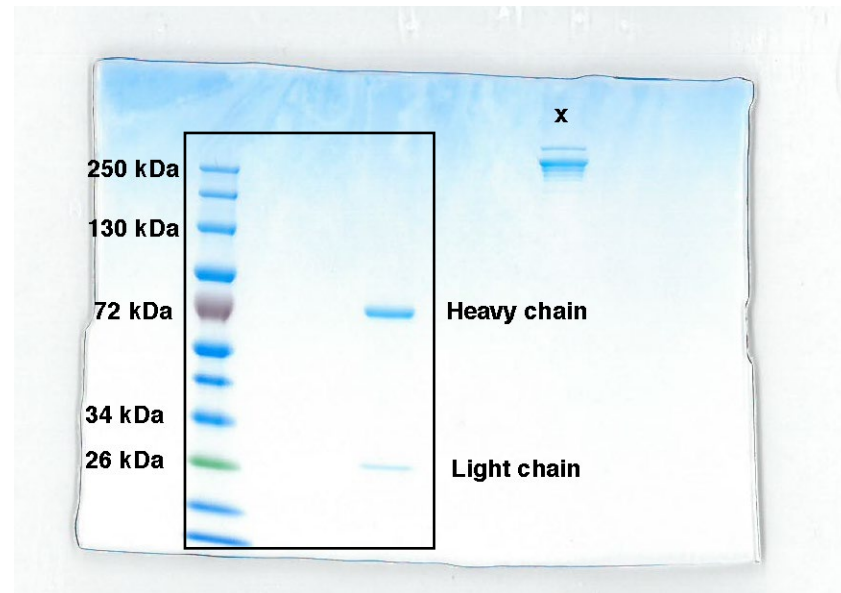

Black rectangle: Area shown in Fig. 1 D

Supplement: S1 Raw images — (PDF) [file pone.0276462.s007.pdf]
