# Supplementary material for: Targeting caveolae to pump bispecific antibody to TGF-β into diseased lungs enables ultra-low dose therapeutic efficacy
Source: PLoS One. 2022 Nov 22;17(11):e0276462. doi: 10.1371/journal.pone.0276462 (PMC9681080; doi:10.1371/journal.pone.0276462)
Supplement: S2 Raw images — (PDF) [file pone.0276462.s008.pdf]

S5 Fig. Raw images of blots Figure 5

P-smad2/3 protein expression

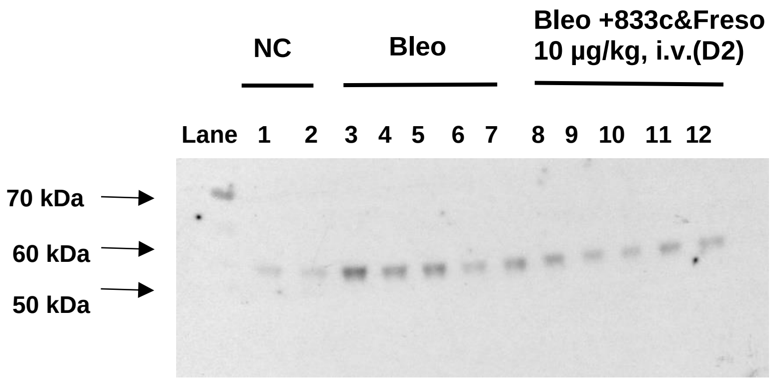

Smad2/3 protein expression

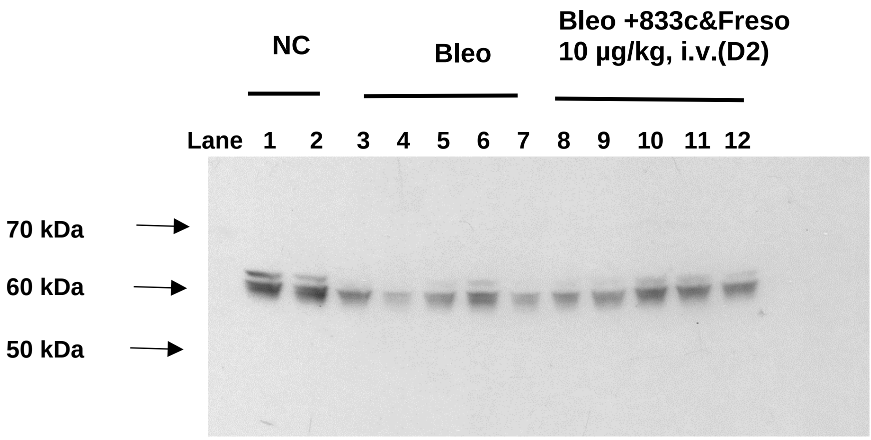

Fibronectin protein expression

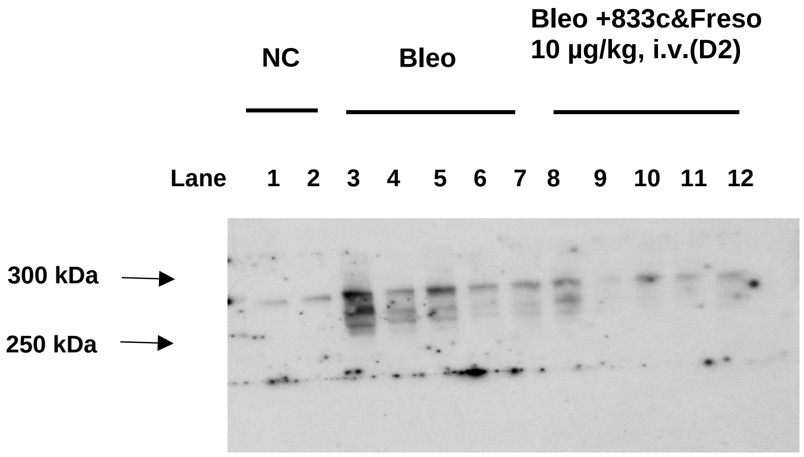

Galectin-3 protein expression

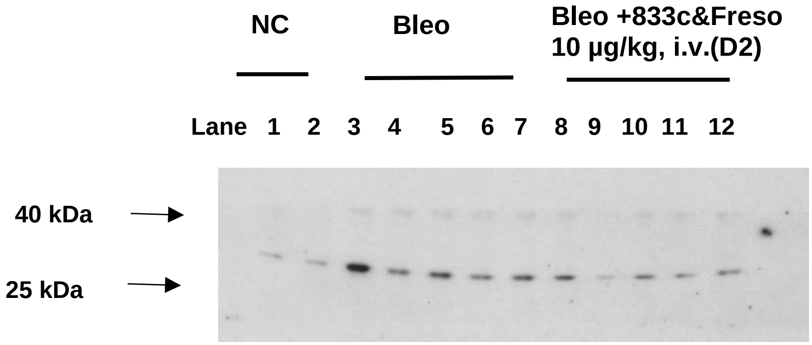

GAPDH control

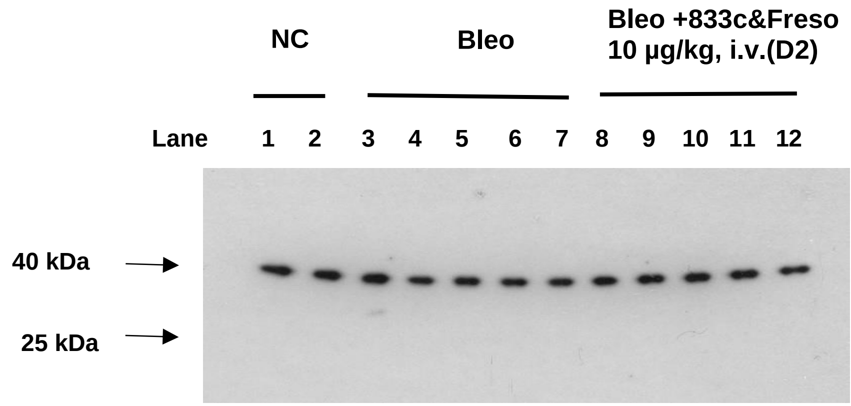

Normal control (NC): *Lane 1, 2*

Bleo: *Lane 3, 4, 5, 6, 7*

Bleo +833c&Freso 10 µg/kg, i.v.(D2): *Lane 8, 9, 10, 11, 12*
