# Supplementary material for: Targeting caveolae to pump bispecific antibody to TGF-β into diseased lungs enables ultra-low dose therapeutic efficacy
Source: PLoS One. 2022 Nov 22;17(11):e0276462. doi: 10.1371/journal.pone.0276462 (PMC9681080; doi:10.1371/journal.pone.0276462)
Supplement: S3 Raw images — (PDF) [file pone.0276462.s009.pdf]

SS5 Fig. Raw images of blots figure S5.

PDGF receptor-β protein expression

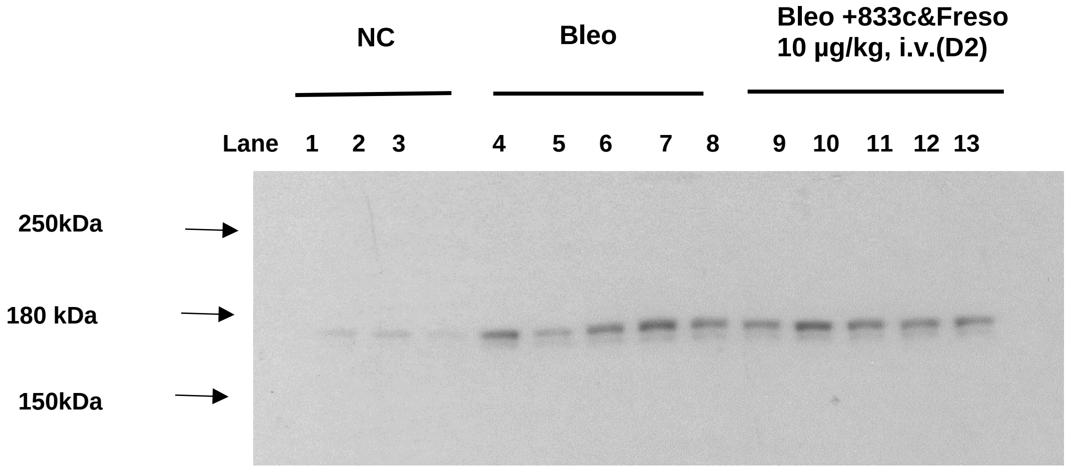

Phospho AKT (Thr-308) protein expression

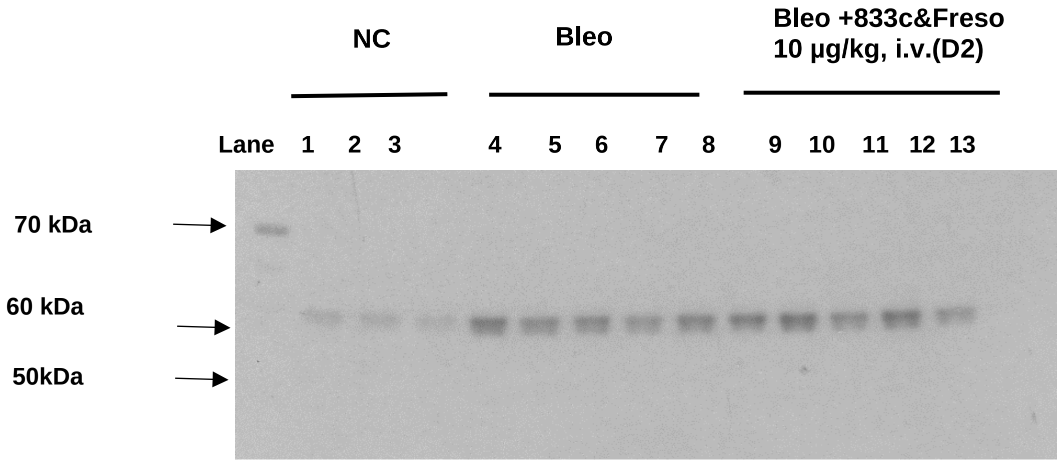

AKT protein expression

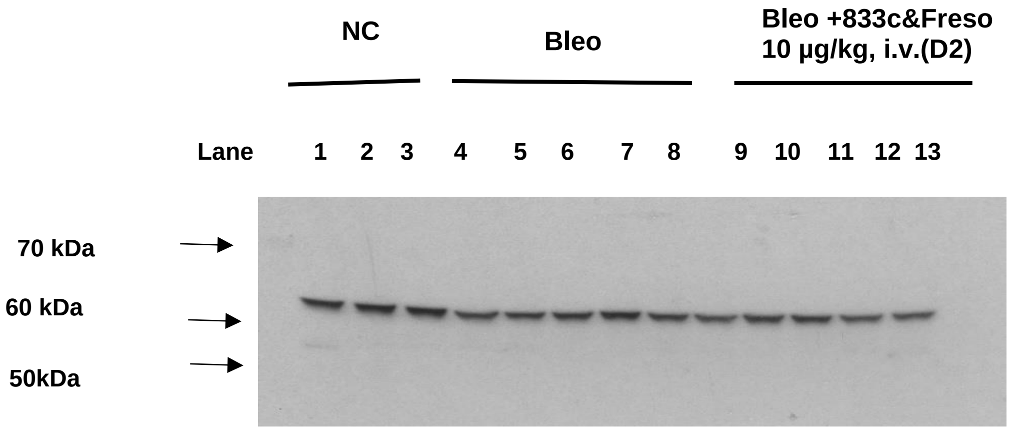

Phospho p-70S6 Kinase (Thr389) protein expression

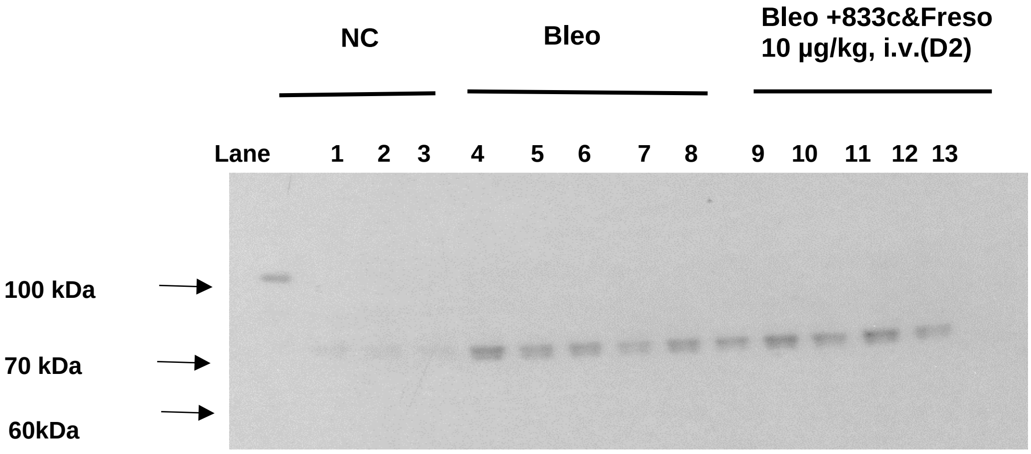

p-70S6 Kinase protein expression

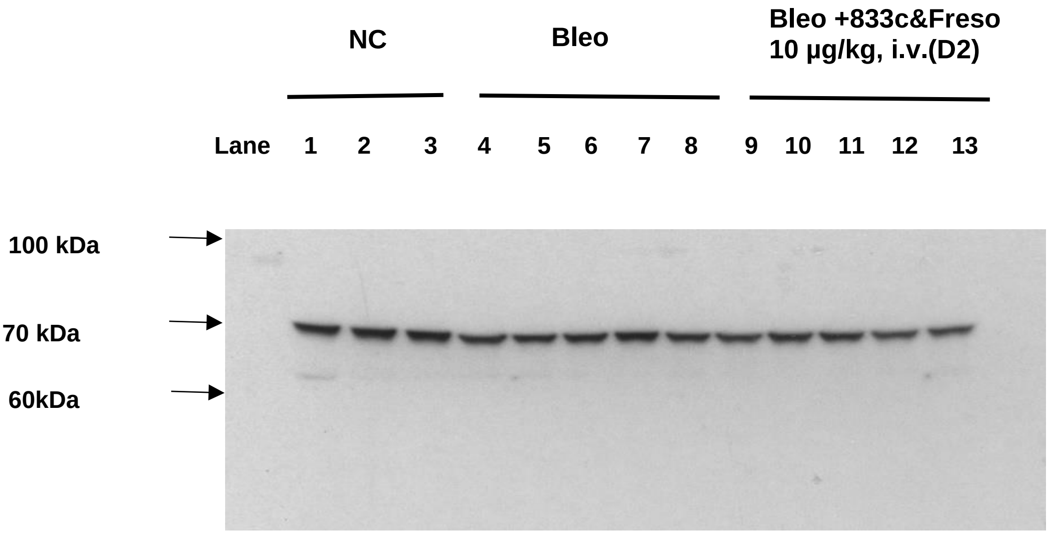

GAPDH control

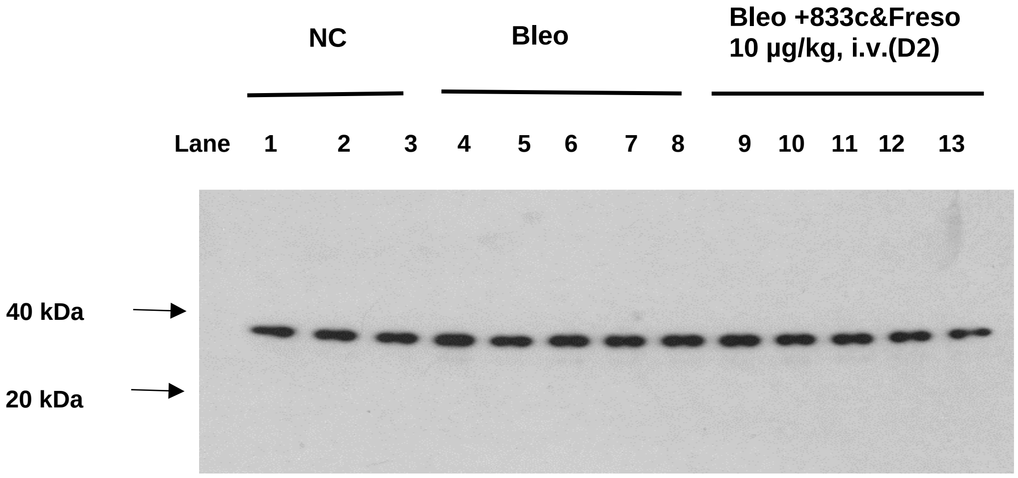

Normal control (NC): *Lane 1, 2, 3*

Bleo: *Lane 4, 5, 6, 7, 8*

Bleo +833c&Freso 10 µg/kg, i.v.(D2): *Lane 9, 10, 11, 12, 13*
